# Supplementary material for: Direct reprogramming of human fibroblasts into insulin-producing cells using transcription factors
Source: Commun Biol. 2023 Mar 24;6:256. doi: 10.1038/s42003-023-04627-2 (PMC10039074; doi:10.1038/s42003-023-04627-2)
Supplement: Supplementary file 2 — Supplementary Information [file 42003_2023_4627_MOESM2_ESM.pdf]

Supplementary Materials
for

Direct reprogramming of human fibroblasts into insulin-producing cells using transcription factors

Running title: Generation of fibroblast-derived insulin producing cells

Marta Fontcuberta-PiSunyer, Ainhoa García-Alamán, Èlia Prades, Noèlia Téllez, Hugo Alves-Figueiredo, Mireia Ramos-Rodríguez, Carlos Enrich, Rebeca Fernandez-Ruiz, Sara Cervantes, Laura Clua, Javier Ramón-Azcón, Christophe Broca, Anne Wojtusciszyn, Nuria Montserrat, Lorenzo Pasquali, Anna Novials, Joan-Marc Servitja, Josep Vidal, Ramon Gomis, Rosa Gasa *

*rgasa@recerca.clinic.cat

This PDF file includes:

Supplementary Figures 1-9, pages 2-10
Supplementary Tables 1-2, pages 11-13

a

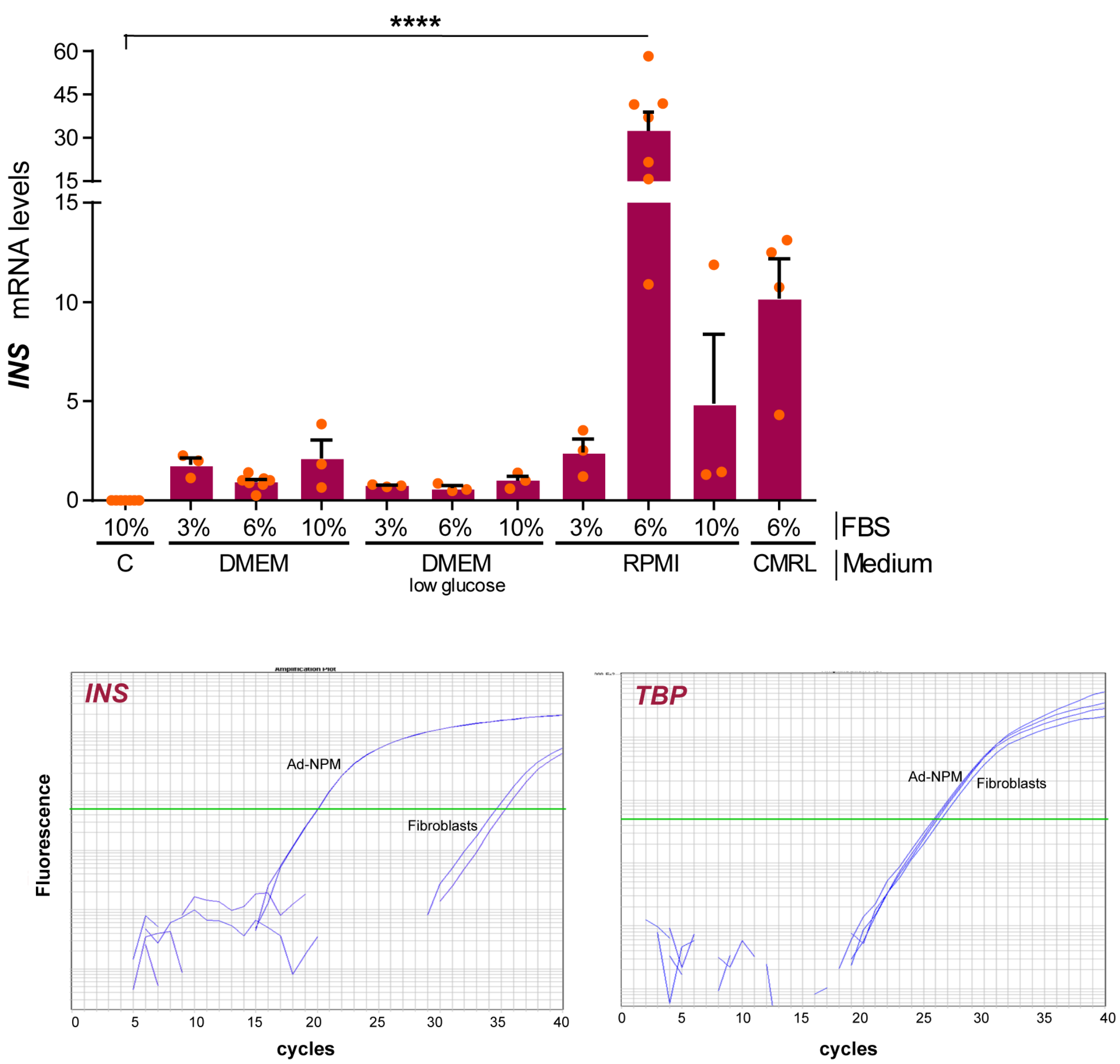

Supplementary Figure 1. Effects of culture media formulation on *INS* gene induction by Ad-NPM in human fibroblasts.

Human fibroblasts (HFF1) were infected with Ad-NPM. After virus removal, cells were cultured in normal fibroblast growth media (DMEM-F12, 4500mg/L glucose and 10% (v/v) Fetal Bovine Serum or FBS) or changed to the culture media and FBS concentrations indicated in the X-axis. Glucose concentrations in DMEM low glucose, RPMI-1640 and CMRL-1066 were 1000mg/L, 2000 mg/L and 1000 mg/L respectively. Seven days later, cells were harvested and RNA extracted to determine *INS* mRNA by qPCR. **(a)** *INS* mRNA levels expressed relative to *TBP*. Values are mean ± SEM of 3-7 independent experiments. **(b)** Representative real time PCR amplification plots for *INS* and *TBP* in parental fibroblasts and in fibroblasts seven days after infection with Ad-NPM. ****, P<0.0001 relative to parental fibroblasts (C) using one-way ANOVA followed by Dunnett's multiple comparison test.

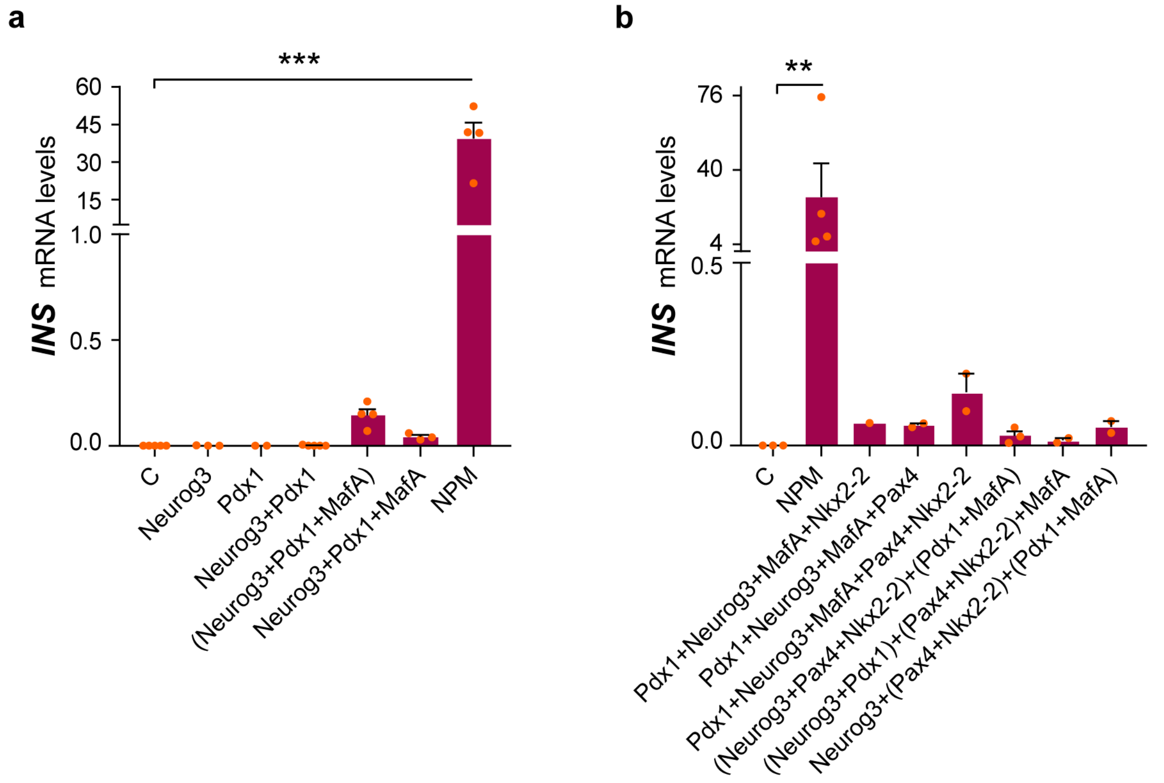

Supplementary Figure 2. Induction of the *INS* gene by different transcription factor combinations in human fibroblasts.

Human fibroblasts (HFF1) were infected with recombinant adenoviruses encoding the transcription factors indicated in the X-axis. Except for NPM, which refers to the polycistronic adenovirus encoding Neurog3, Pdx1 and Mafa, all other adenoviruses encoded individual transcription factors. Adenoviruses were added either sequentially (order of addition is shown left to right) or simultaneously (shown in parenthesis). The interval between subsequent infections was two days. **(a)** qPCR for *INS* seven days after initial infection. **(b)** qPCR of *INS* ten days after initial infection. Expression levels are calculated relative to *TBP*. Data are mean ± SEM for n=2-5 independent experiments. **,P<0.01; ***,P<0.001 relative to parental fibroblasts (C) using one-way ANOVA followed by Dunnett's multiple comparison test.

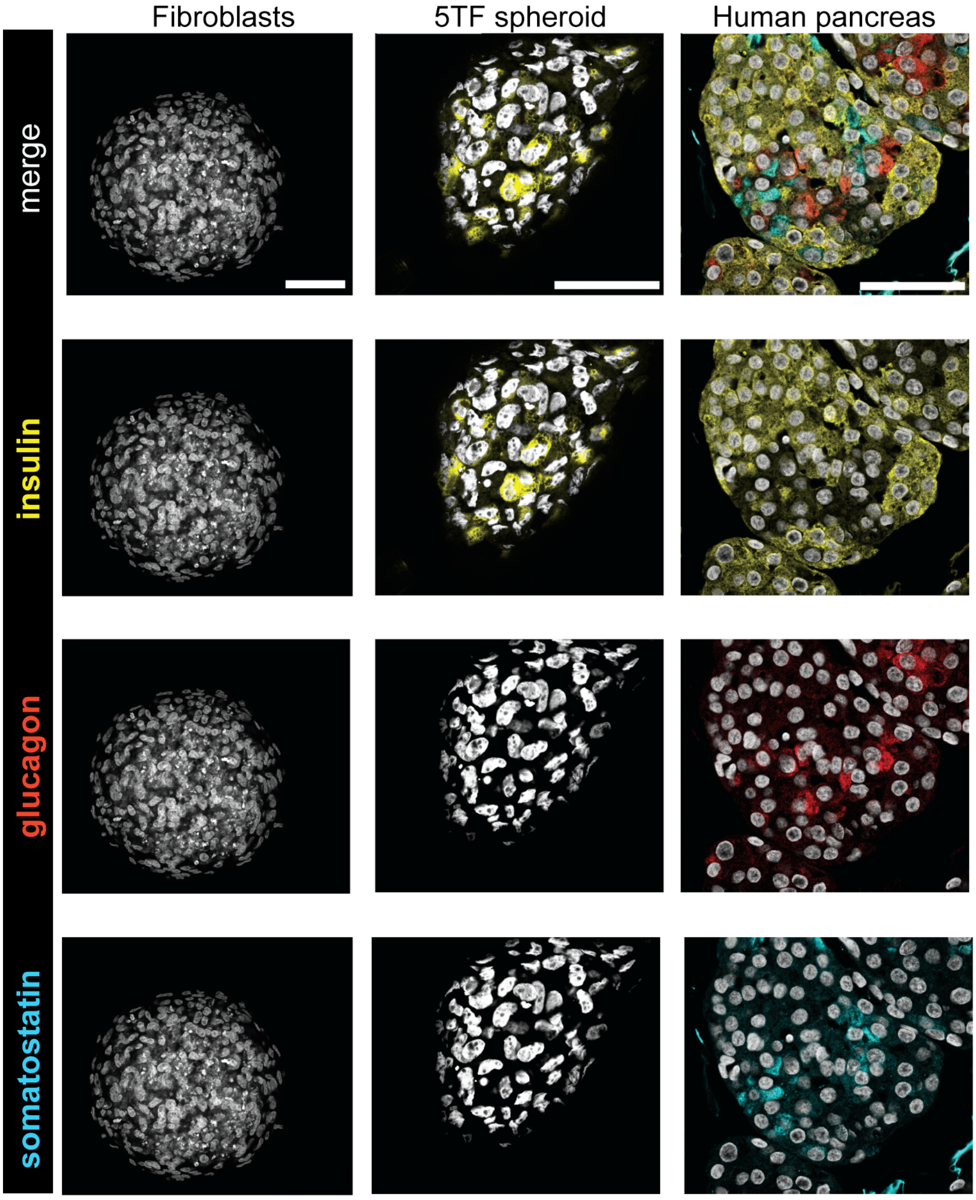

Supplementary Figure 3. Staining for islet hormones in 5TF cell spheroids. Representative confocal images of 5TF cell spheroids collected at day 11 of the 5TF-3D reprogramming protocol and immunostained with the indicated antibodies. Insulin is shown in yellow, glucagon in red and somatostatin in light blue. In the merge image, nuclei are shown in white (marked with Hoechst). Spheroids consisting of parental fibroblasts and fixed human pancreas are shown as negative and positive control, respectively. Scale bars, 50 μm.

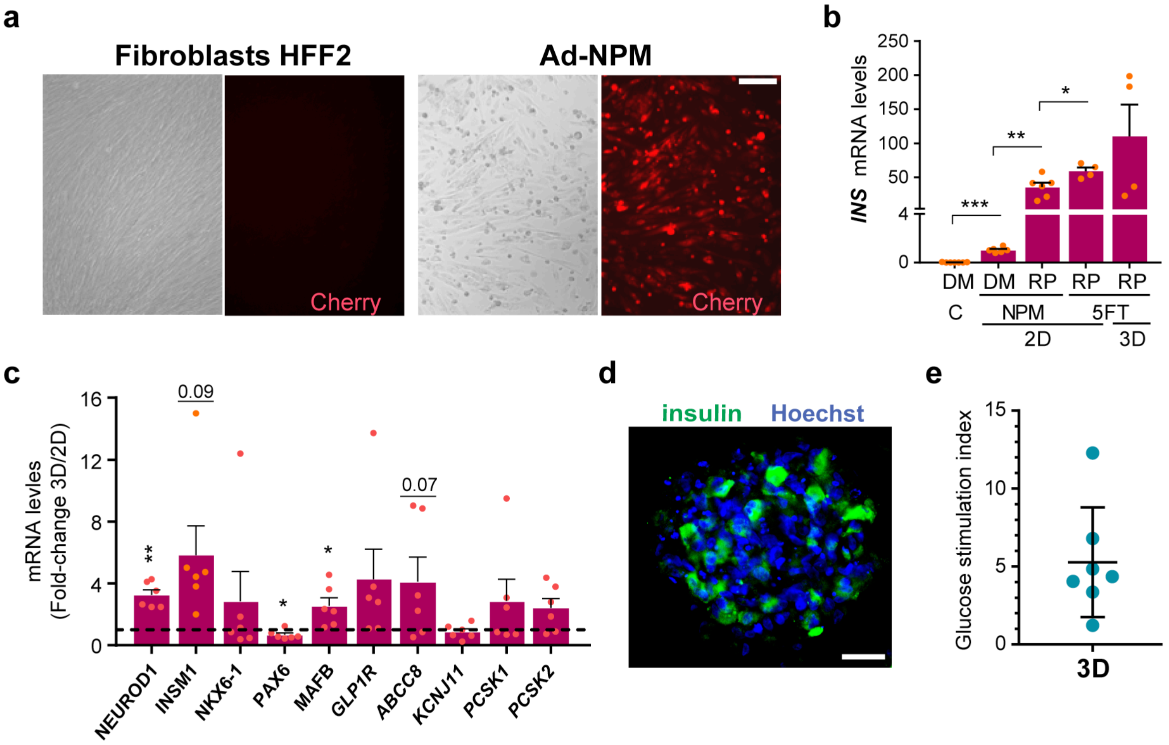

Supplementary Figure 4. Validation of the 5TF-3D protocol in a second HFF preparation.

Human foreskin fibroblasts (HFF2) were subjected to transcription factor-based reprogramming toward insulin-producing cells. **(a)** Bright field and Cherry immunofluorescence images from control (parental) fibroblasts and from fibroblasts three days after infection with Ad-NPM. Scale bar, 100 μm. **(b)** qPCR for *INS* in HFF2 ten days after infection with Ad-NPM alone and cultured under 2D conditions in DMEM/10%FBS (DM) or RPMI-1640/6% FBS media (RP) (n=6), or after the 5TF reprogramming protocol and cultured in RP media under 2D or 3D conditions (n=4). Expression levels were calculated relative to *TBP*. Note that changes in *INS* gene expression in the different reprogramming conditions follow a similar trend as observed with HFF1 fibroblasts. **(c)** qPCR of the indicated genes in HFF2-derived 5TF cell spheroids (n=6). Transcript levels are expressed relative to spheroids maintained in 2D culture throughout the 10-day protocol (given the value of 1). **(d)** Representative immunofluorescence image showing insulin (using antibody ab2 against C-peptide) in green and nuclei in blue (marked with Hoechst). Scale bar, 100 μm. **(e)** Glucose stimulation Index (ratio between insulin secreted at 20mM glucose vs. 2mM glucose) of 5TF cells spheroids (n=7, from 3 reprogramming experiments). Data are mean ± SEM. *, P< 0.05; **, P< 0.01; ***, P< 0.001 between indicated conditions in **(b)** using unpaired t-test and relative to 2D in **(c)** using one-sample test.

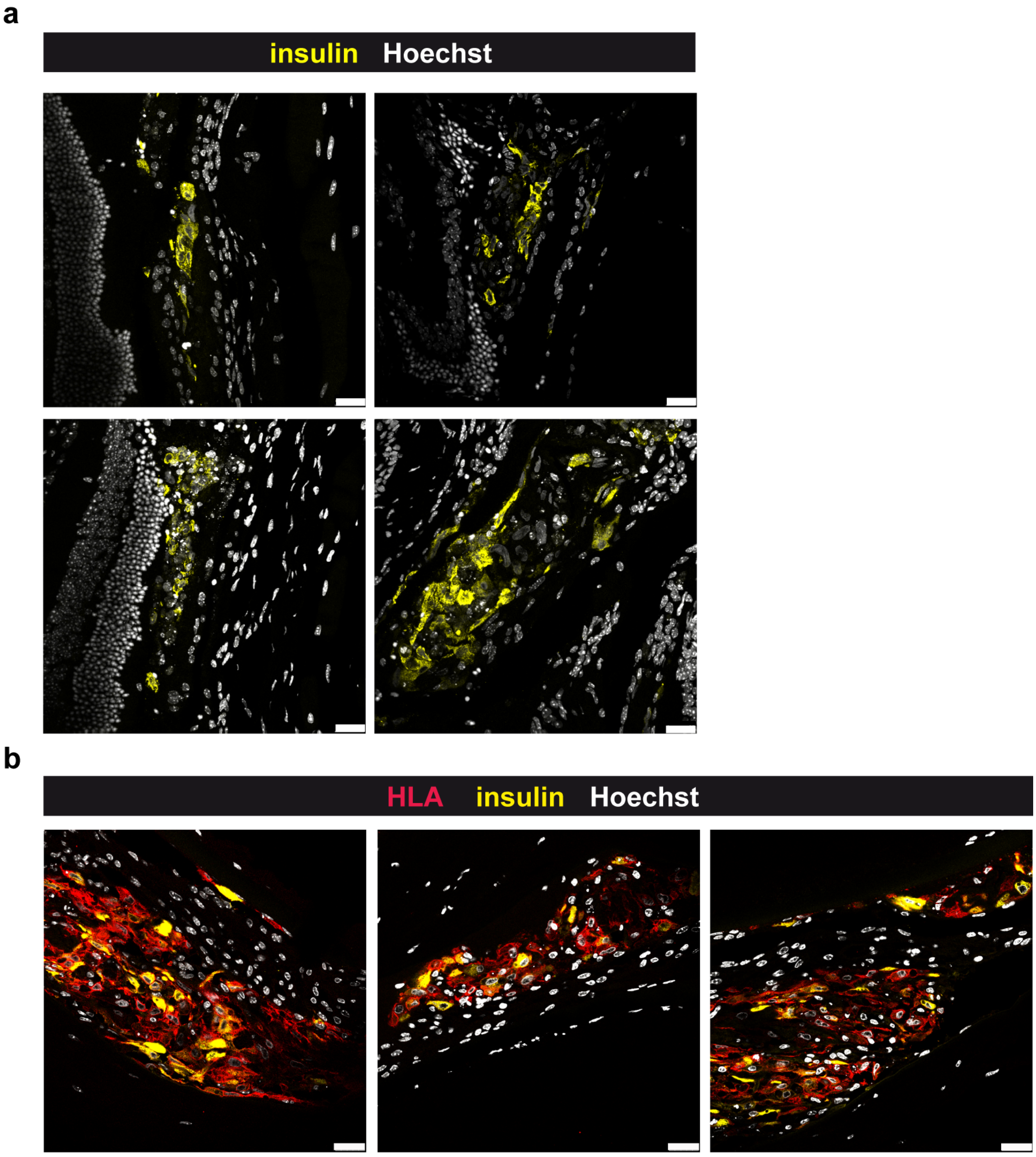

Supplementary Figure 5. Identification of insulin-positive cells in 10-day eye grafts. Eye grafts were harvested ten days following transplantation of 5TF cell spheroids into the anterior chamber of the eye of normoglycemic NSG mice. After fixation, grafts were immunostained with the indicated antibodies. Figure shows representative confocal images of different grafts. Insulin is shown in yellow **(a,b)** and HLA (to identify human cells) in red **(b)**. Nuclei were marked with Hoechst and are shown in white in **(a,b)**. Scale bar, 25 μm.

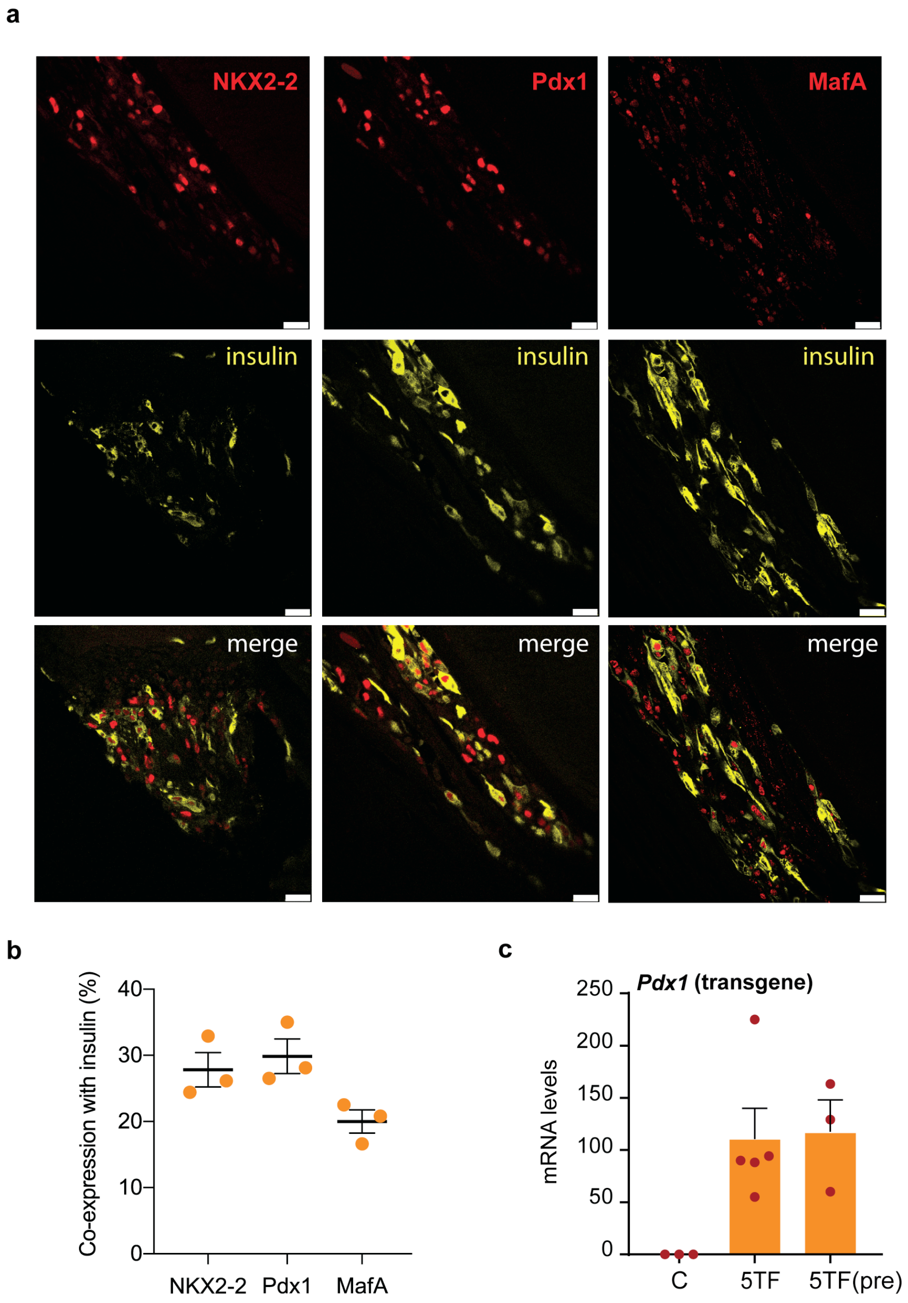

Supplementary Figure 6. Expression of the reprogramming transcription factors in 10-day grafts.

Eye grafts were harvested ten days following transplantation of 5TF cell spheroids into the anterior chamber of the eye of normoglycemic NSG mice. **(a)** Representative confocal immunofluorescence images showing insulin staining in yellow and staining for the indicated transcription factors in red. Note that antibodies do not differentiate endogenous protein from virally generated exogenous transcription factors. Scale bars, 25 μm. **(b)** Percentage of cells double-stained for insulin and the indicated transcription factors (relative to total insulin-positive cells). Each dot corresponds to one eye graft. Lines represent mean ± SEM. **(c)** qPCR for the *Pdx1* transgene in eye grafts transplanted with parental fibroblasts (n=3) or 5TF cell spheroids (n=5). *Pdx1* transgene levels in 5TF cell spheroids before transplantation (n=3) are shown as reference. Expression levels are calculated relative to *TBP* and are mean ± SEM.

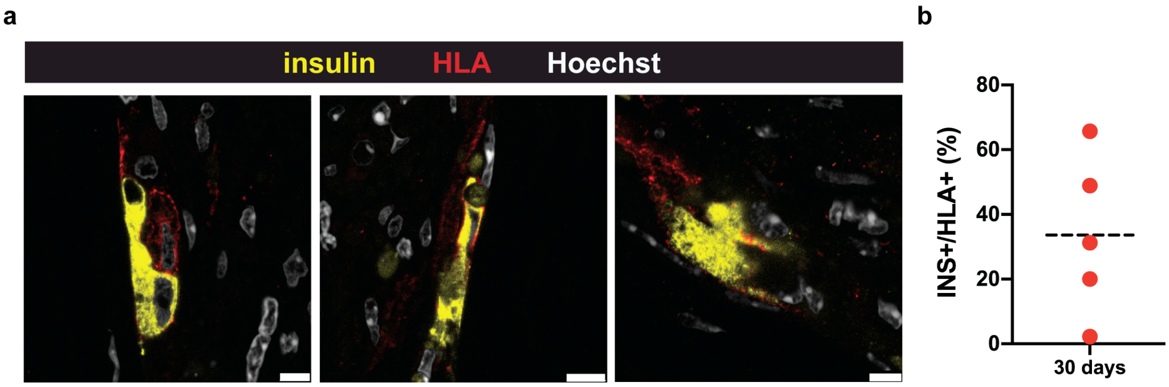

Supplementary Figure 7. Identification of insulin-positive cells in 30-day grafts. Eye grafts were harvested thirty days following transplantation of 5TF cell spheroids into the anterior chamber of the eye of normoglycemic NSG mice. **(a)** Representative confocal immunofluorescence images showing insulin staining in yellow and HLA staining (to identify human cells) in red. Nuclei are marked with Hoechst in white. Scale bars, 5 μm. **(b)** Percentage of cells exhibiting double-positive staining for insulin and HLA (relative to total HLA+ cells) in five different eye grafts. Each dot corresponds to one graft. Horizontal dotted line represents the median.

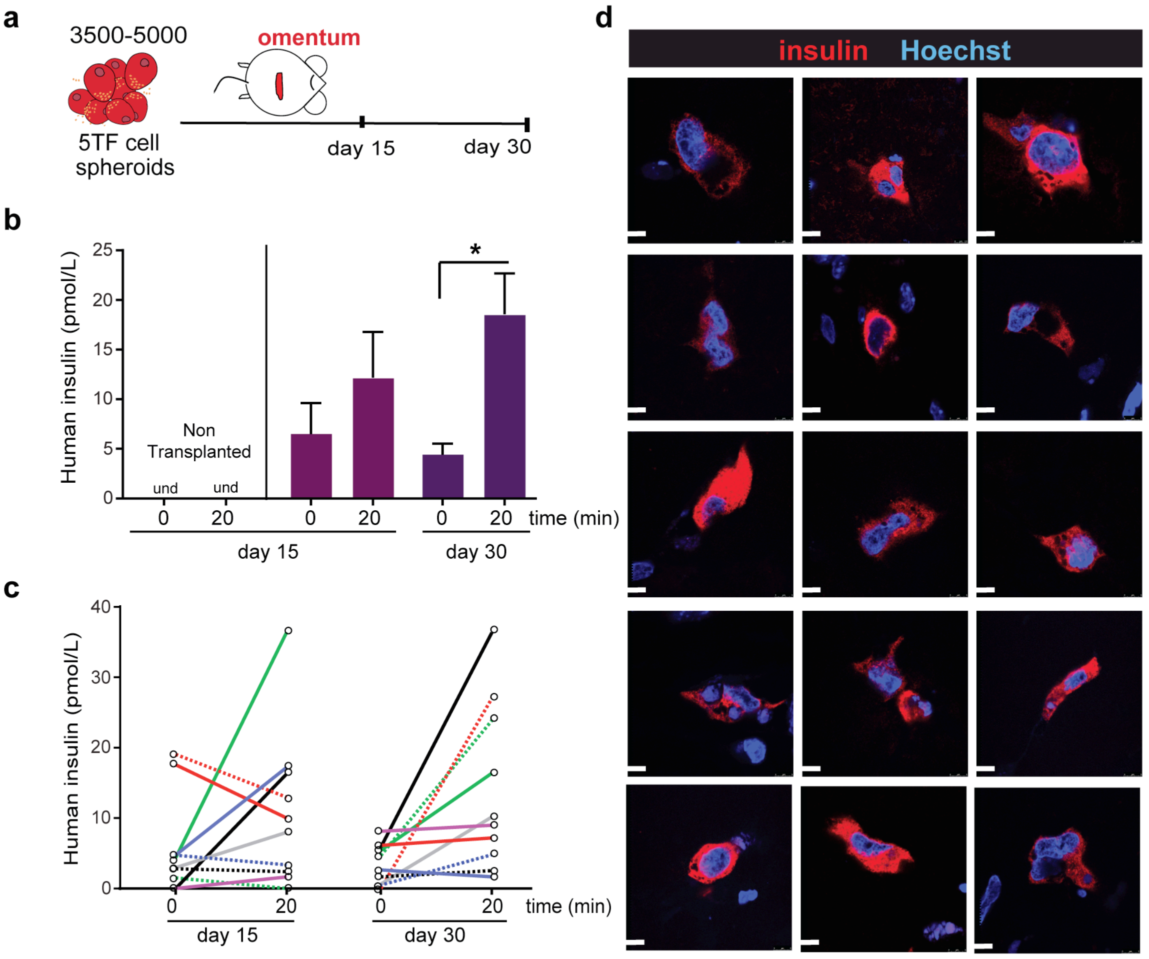

Supplementary Figure 8. Transplantation of 5TF cell spheroids in the omentum.
5TF cell spheroids were transplanted in the omentum of normoglycemic NSG mice. **(a)** Schematic illustration depicting the experimental design. **(b)** Glucose-induced insulin secretion was studied at days 15 and 30 after transplantation. ELISA determination of human insulin in plasma in mice transplanted with 5TF cell spheroids (n=10) at basal and 20 minutes after an intraperitoneal glucose injection. Data are presented as mean ± SEM. *, P< 0.05 between indicated conditions using an unpaired t-test. Human insulin levels in non-transplanted mice (n=6) were undetectable. **(c)** Individual insulin secretory responses. Each mouse is depicted with a distinct color or line style. **(d)** Examples of confocal pictures of cells stained positively for insulin (in red) in three distinct omentum grafts that were harvested 30 days after transplantation. Nuclei are marked with Hoechst in blue. Scale bars, 5 μm.

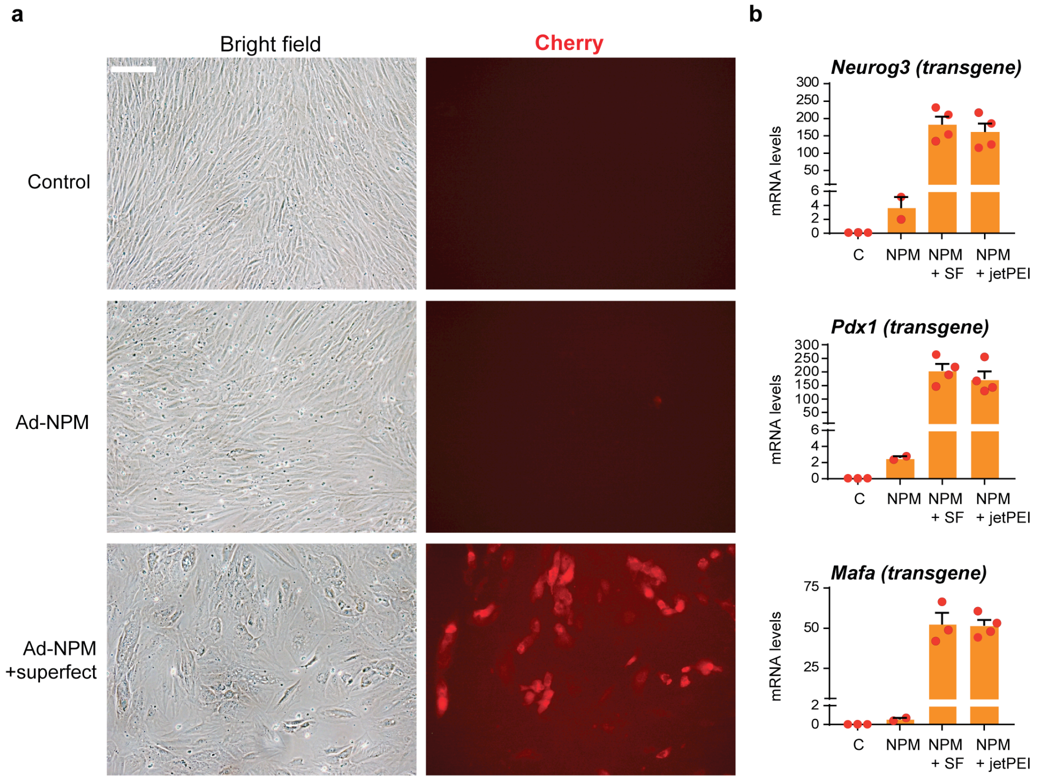

Supplementary Figure 9. Improvement of adenoviral infection efficiency in human fibroblasts by including DNA transfection reagents.

Human fibroblasts (HFF1) were infected with a polycistronic recombinant adenovirus encoding Neurog3, Pdx1, Mafa and a Cherry reporter protein (Ad-NPM), in the presence or absence of the transfection reagents Superfect and jetPEI. **(a)** Bright field and Cherry immunofluorescence images from control (parental) fibroblasts and HFF1 fibroblasts three days after infection with Ad-NPM alone or Ad-NPM + Superfect. **(b)** qPCR of the transgenes three days after infection with Ad-NPM in the presence or absence of the indicated transfection reagent**.** Data are presented as the mean ± SEM for n= 2 for NPM alone and n=3-4 for the other conditions. Scale bar, 100 μm.

Supplementary Table 1. List of oligonucleotides used for real time PCR.

Gene	Species	Primer ID	Primer sequence (5' to 3')
ABCC8	human	*Forward*	AGACCCTCATGAACCGACAG
ABCC8	human	*Reverse*	GGCTCTGTGGCTTTTCTCTC
CD29	human	*Forward*	CAAAGGAACAGCAGAGAAGC
CD29	human	*Reverse*	ATTGAGTAAGACAGGTCCATAAGG
CD49a	human	*Forward*	GCTGGCTCCTCACTGTTGTT
CD49a	human	*Reverse*	CTCCATTTGGGTTGGTGACT
CHGB	human	*Reverse*	TTGGATGTCCTCCTCCCCTG
CHGB	human	*Forward*	CGCCAAGTCCTGAAGACGA
COL1A1	human	*Forward*	CTCGAGGTGGACACCACCCT
COL1A1	human	*Reverse*	CAGCTGGATGGCCACATCGG
FOXF2	human	*Forward*	CTACTTGCACCAGAACGCTC
FOXF2	human	*Reverse*	CGCAGGGCTTAATATCCTGACA
GCG	human	*Forward*	CACAGGGCACATTCACCAG
GCG	human	*Reverse*	TCTGGGAAATCTCGCCTTCC
GIPR	human	*Forward*	ACAATGTGAGAACCCAGAGAAG
GIPR	human	*Reverse*	CGCCTGAACAAACTCAAGATG
GLP1R	human	*Forward*	TTCTCTGCTCTGGTTATCGCC
GLP1R	human	*Reverse*	GGATGCAAACAGGTTCAGGT
HNF1B	human	*Forward*	ACCAAGCCGGTCTTCCATACT
HNF1B	human	*Reverse*	GGTGTGTCATAGTCGTCGCC
INS	human	*Forward*	GCAGCCTTTGTGAACCAACA
INS	human	*Reverse*	TTCCCCGCACACTAGGTAGAGA
INSM1	human	*Forward*	TTTTGGAACCCCCACTTTTAC
INSM1	human	*Reverse*	CGAGACCAGACCGCATTT
KCNJ11	human	*Forward*	TGTGTCACCAGCATCCACTC
KCNJ11	human	*Reverse*	CACTTGGACCTCAATGGAGAA
LDHA	human	*Forward*	GCAAGAGGGAGAAAGCCGTC
LDHA	human	*Reverse*	CTTCCAAGCCACGTAGGTCA
LHX9	human	*Forward*	TGCCAAGGACGGTAGCATTT
LHX9	human	*Reverse*	GCAGCTCAGGTGGTAGACAG
MafA	mouse	*Forward*	CTTCAGCAAGGAGGAGGTCA
MafA	mouse	*Reverse*	TTGTACAGGTCCCGCTCTTT
MAFB	human	*Forward*	AACTTTGTCTTGGGGCACAC
MAFB	human	*Reverse*	GGGACCTCTCGGTTCTCTCT
NCAM 1	human	*Forward*	CCTCCCAGCCAGCAGATTAC
NCAM1	human	*Reverse*	CTCTCCAACGCTGATCTCCC
NEUROD1	human	*Forward*	AGGAAGAAGAGGAAGAGGAGGATG
NEUROD1	human	*Reverse*	TTGGTGGTGGGTTGGGATAAG
NEUROG3	human	*Forward*	AGACGACGCGAAGCTCACC
NEUROG3	human	*Reverse*	AAGCCAGACTGCCTGGGCT
Neurog3	mouse	*Forward*	CCCACCTAGCCCCACTCTCATACC

Neurog3	mouse	*Reverse*	CGCCGGCTTCTTCGCTGTTT
NKX2.2	human	*Forward*	CTTCTACGACAGCAGCGACAACCCG
NKX2.2	human	*Reverse*	CCTTGGAGAAAAGCACTCGCCGCTTT
NKX6-1	human	*Forward*	ACACGAGACCCACTTTTTCCG
NKX6-1	human	*Reverse*	GCCCCGCCAAGTATTTTGTT
OAT	human	*Forward*	CGTAAGTGGGGCTATACCGT
OAT	human	*Reverse*	CTGGTTGGGTCTGTGGAACT
PAX4	human	*Forward*	AGCAGAGGCACTGGAGAAAGAGTT
PAX4	human	*Reverse*	CAGCTGCATTTCCCACTTGAGCTT
PAX6	human	*Forward*	AGACACAGCCCTCACAAACA
PAX6	human	*Reverse*	ATCATAACTCCGCCCATTCA
PCSK1	human	*Forward*	AAGCAAACCCAAATCTCACCTGGC
PCSK1	human	*Reverse*	TCACCATCAAGCCTGCTCCATTCT
PCSK2	human	*Forward*	CCGGGTTCCTCTTCTGTGTC
PCSK2	human	*Reverse*	AGCAAAGGGAAGCTTTCGGA
PDX1	human	*Forward*	CCCATGGATGAAGTCTACC
PDX1	human	*Reverse*	GTCCTCCTCCTTTTTCCAC
Pdx1	mouse	*Forward*	CCCCAGTTTACAAGCTCGCT
Pdx1	mouse	*Reverse*	CTCGGTTCCATTCGGGAAAGG
PRRX1	human	*Forward*	CGAGAGTGCAGGTGTGGTTT
PRRX1	human	*Reverse*	GAGCAGGACGAGGTACGATG
PTPRN (IA2)	human	*Reverse*	GGTGGAGGATGGTGTCAAGC
PTPRN (IA2)	human	*Forward*	AAGCTCCGCACCAGAAAGTC
SMAD3	human	*Forward*	CATCGAGCCCCAGAGCAATA
SMAD3	human	*Reverse*	TTTGGAGAACCTGCGTCCAT
SST	human	*Forward*	CAGTTTCTGCAGAAGTCCCTG
SST	human	*Reverse*	AATTCTTGCAGCCAGCTTTGC
TBP	human	*Forward*	ATCCCTCCCCCATGACTCCCATG
TBP	human	*Reverse*	ATGATTACCGCAGCAAACCGC
Tbp	mouse	*Forward*	ACCCTTCACCAATGACTCCTATG
Tbp	mouse	*Reverse*	ATGATGACTGCAGCAAATCGC
TBP	human(specific)	*Forward*	TGTGCTCACCCACCAACAAT
TBP	human(specific)	*Reverse*	ACGTCGTCTTCCTGAATCCC
TWIST2	human	*Forward*	CGCAAGTGGAATTGGGATGC
TWIST2	human	*Reverse*	CGATGTCACTGCTGTCCCTT

Supplementary Table 2. Summary of 5TF cell spheroid transplantation experiments.

Site	Mouse ID	Cells	Reprog. ID	Cell # (x 10^6)	Transp. days	Plasma Human INS (pmol/L)		INS+ cells
						t0	t20	
Subcut.	S1	5TF	ID48	3,6	15	1,249	3,955	
					30	<0.001	2,262	ND
Subcut.	S2	5TF	ID48	2,7	15	1,318	6,315	
					30	4,955	9,542	NA
Subcut.	S3	5TF	ID50	3,6	15	1,693	11,485	
					30	1,61	16,378	NA
Kidney	K1	5TF	HFF140	6	15	13,706	36,934	
					30	4,517	35,678	NA
Kidney	K2	5TF	HFF147	5,4	15	6,479	<0.001	
					30	3,381	7,127	NA
Kidney	K3	5TF	HFF147	4,8	15	<0.001	0,957	
					30	3,381	2,705	ND
Omentum	Om1	5TF	ID50	3,6	15	4,355	36,406	
					30	5,424	16,390	NA
Omentum	Om2	5TF	ID54	4	15	1,521	<0.001	
					30	4,667	23,863	NA
Omentum	Om3	5TF	ID54	4	15	<0.001	16,286	
					30	5,869	36,343	NA
Omentum	Om4	5TF	AG27	5	15	19,064	12,98	
					30	<0.001	27,016	ND
Omentum	Om5	5TF	AG27	5	15	2,966	7,987	
					30	0,646	10,167	NA
Omentum	Om6	5TF	MF20	5	15	<0.001	1,646	
					30	8,126	8,994	NA
Omentum	0m7	5TF	AG27	5	15	17,592	10,008	
					30	6,125	7,188	YES
Omentum	Om8	5TF	AG36	4,8	15	2,789	2,387	
					30	1,658	2,54	YES
Omentum	Om9	5TF	AG36	4,8	15	4,712	3,379	
					30	0,499	4,795	YES
Omentum	Om10	5TF	AG36	4,8	15	4,746	17,218	
					30	2,637	1,658	NA
Non-transplanted	CTRL1	no	_	0		<0.001	<0.001	_
Non-transplanted	CTRL2	no	_	0		<0.001	<0.001	_
Non-transplanted	CTRL3	no	_	0		<0.001	<0.001	_
Non-transplanted	CTRL4	no	_	0		<0.001	<0.001	_
Non-transplanted	CTRL5	No	_	0		<0.001	<0.001	_
Non-transplanted	CTRL6	No	_	0		<0.001	<0.001	_

Reprog ID: Reprogramming experiment identification code
Transp. days: follow-up period after transplant (15 and 30 days)
t0, t20: before and 20 minutes after an intraperitoneal glucose injection
INS+ cells: detection of insulin-positive cells by immunostaining in harvested grafts. NA: not available; ND: not detected
