# Supplementary material for: Direct reprogramming of human fibroblasts into insulin-producing cells using transcription factors
Source: Commun Biol. 2023 Mar 24;6:256. doi: 10.1038/s42003-023-04627-2 (PMC10039074; doi:10.1038/s42003-023-04627-2)
Supplement: Supplementary file 3 — Description of Additional Supplementary Files [file 42003_2023_4627_MOESM3_ESM.pdf]

Description of Additional Supplementary Files

• File name: Supplementary Data 1

Description: List of differentially expressed genes in 5TF cell spheroids as compared to fibroblasts with an adjusted p-value <0.05 and fold-change (FC)>2.

• File name: Supplementary Data 2

Description: The source data behind the graphs in the paper

• File name: Supplementary Video 1

Description: Video shows changes in fluorescence of an isolated 5TF cell in response to high glucose (20mM) and high potassium (KCl 30mM). Cell had been preloaded with the calcium indicator Fluo-2.

Video was recorded using a Leica TCS SPE confocal microscope with an incubation chamber set at 37oC, and a 40X oil immersion objective.

• File name: Supplementary Video 2

Description: Video shows fluorescence of parental fibroblasts HFF1 in response to high glucose (20mM) and high potassium (KCl 30mM). Cells had been pre-loaded with the calcium indicator Fluo-2. Video was recorded using a Leica TCS SPE confocal microscope with an incubation chamber set at 37oC, and a 40X oil immersion objective.
