# Supplementary material for: Direct reprogramming of human fibroblasts into insulin-producing cells using transcription factors
Source: Commun Biol. 2023 Mar 24;6:256. doi: 10.1038/s42003-023-04627-2 (PMC10039074; doi:10.1038/s42003-023-04627-2)
Supplement: Supplementary file 8 — Reporting Summary [file 42003_2023_4627_MOESM8_ESM.pdf]

nature portfolio

Corresponding author(s): Rosa Gasa

Last updated by author(s): 2022/2/5

Reporting Summary

Nature Portfolio wishes to improve the reproducibility of the work that we publish. This form provides structure for consistency and transparency in reporting. For further information on Nature Portfolio policies, see our Editorial Policies and the Editorial Policy Checklist.

Statistics

For all statistical analyses, confirm that the following items are present in the figure legend, table legend, main text, or Methods section.

n/a	Confirmed	
☐	☒	The exact sample size (*n*) for each experimental group/condition, given as a discrete number and unit of measurement
☒	☐	A statement on whether measurements were taken from distinct samples or whether the same sample was measured repeatedly
☐	☒	The statistical test(s) used AND whether they are one- or two-sided<br>*Only common tests should be described solely by name; describe more complex techniques in the Methods section.*
☒	☐	A description of all covariates tested
☒	☐	A description of any assumptions or corrections, such as tests of normality and adjustment for multiple comparisons
☐	☒	A full description of the statistical parameters including central tendency (e.g. means) or other basic estimates (e.g. regression coefficient) AND variation (e.g. standard deviation) or associated estimates of uncertainty (e.g. confidence intervals)
☒	☐	For null hypothesis testing, the test statistic (e.g. *F*, *t*, *r*) with confidence intervals, effect sizes, degrees of freedom and *P* value noted<br>*Give P values as exact values whenever suitable.*
☒	☐	For Bayesian analysis, information on the choice of priors and Markov chain Monte Carlo settings
☒	☐	For hierarchical and complex designs, identification of the appropriate level for tests and full reporting of outcomes
☒	☐	Estimates of effect sizes (e.g. Cohen's *d*, Pearson's *r*), indicating how they were calculated

Our web collection on statistics for biologists contains articles on many of the points above.

Software and code

Policy information about availability of computer code

Data collection	qRT-PCR data was collected using the sds 2.4 software (ABI7900, Applied Biosystems). ELISA data was collected using Gen5 v1.10 (Biotek Synergy). Confocal image acquisition was performed with Leica LAS AF v2.7.3.9723. Conventional transmission electron microscopy (TEM) images were acquired using a JEOL-1010 electron microscopy equipped with an SC1000 ORIUS-CCD digital camara (Gatan). RNA sequencing data: Illumina NextSeq2000.
Data analysis	Data were analysed with Graphpad 6 and Microsoft Excel. Image J was used for immunoimaging analysis. RNA seq data: DESeq2 R package v.1.36.0, ClusterProfiler R package v.4.4.1,fgsea R package v.1.22.0. All information about software is reported in the materials and methods section of the manuscript.

For manuscripts utilizing custom algorithms or software that are central to the research but not yet described in published literature, software must be made available to editors and reviewers. We strongly encourage code deposition in a community repository (e.g. GitHub). See the Nature Portfolio guidelines for submitting code & software for further information.

Data

Policy information about availability of data

All manuscripts must include a data availability statement. This statement should provide the following information, where applicable:

- Accession codes, unique identifiers, or web links for publicly available datasets
- A description of any restrictions on data availability
- For clinical datasets or third party data, please ensure that the statement adheres to our policy

RNA sequencing data are deposited in the Gene Expression Omnibus database under accession code GSE210075. Other data supporting the findings of this manuscript are available from the authors upon reasonable request.

Field-specific reporting

Please select the one below that is the best fit for your research. If you are not sure, read the appropriate sections before making your selection.

☒ Life sciences ☐ Behavioural & social sciences ☐ Ecological, evolutionary & environmental sciences

For a reference copy of the document with all sections, see nature.com/documents/nr-reporting-summary-flat.pdf

Life sciences study design

All studies must disclose on these points even when the disclosure is negative.

Sample size

No statistical method was used to predetermine sample size. Sample size was chosen to ensure adequate power based on the available literature and protocols in the field.

Data exclusions

No data were excluded.

Replication

For in vitro experiments: more than three biological replicates (on different dates) and two technical replicates were performed to ensure data reproducibility. All replications were successful. The exact number of replicates for each experiment is indicated in its corresponding figure legend.
For in vivo experiments: n=3 mice were used for transplantation in the kidney and subcutaneous space. At least n=8 mice were used for ACE and omentum sites. Cells used for transplantation experiments were from at least 2 independent reprogramming experiments.

Randomization

Primary fibroblasts were randomly assigned to experimental and control (untreated) groups. Mice of similar age and weight were randomly assigned to experimental and control groups.

Blinding

Investigators were not blinded to group allocations during data collections and analysis. Each round of biological replicates contained all treatment groups.

Reporting for specific materials, systems and methods

We require information from authors about some types of materials, experimental systems and methods used in many studies. Here, indicate whether each material, system or method listed is relevant to your study. If you are not sure if a list item applies to your research, read the appropriate section before selecting a response.

Materials & experimental systems

n/a	Involved in the study	
☐	☒	Antibodies
☐	☒	Eukaryotic cell lines
☒	☐	Palaeontology and archaeology
☐	☒	Animals and other organisms
☐	☒	Human research participants
☒	☐	Clinical data
☒	☐	Dual use research of concern

Methods

n/a	Involved in the study	
☒	☐	ChIP-seq
☒	☐	Flow cytometry
☒	☐	MRI-based neuroimaging

Antibodies

Antibodies used

Primary antibodies used were: guinea pig anti-insulin (A0564, DAKO, 1:500); mouse anti-C-peptide (Hybridoma Bank; 1:40); goat anti-vimentin (Sigma, 1:200); mouse anti-HLA (Abcam, 1:100); mouse anti-Nkx-2.2 (Hybridoma Bank, 1:400); guinea pig anti-Pdx1 (Abcam 1:500); rabbit anti-MafA (Novus Biologicals 1:75); and mouse anti-KCNJ11, mouse anti-PTPRN, mouse anti-NCAM1 (Santa Cruz, 1:50). Secondary antibodies were coupled to Alexa 555 (Molecular Probes), Alexa 488,647 (Jackson Immunoresearch). Normal goat and donkey serum were used (Jackson Immunoresearch).

Validation

All antibodies used were validated by the commercial source for the applications used in this study.

Eukaryotic cell lines

Policy information about cell lines

Cell line source(s)

HFF1 fibroblasts were prepared from a child foreskin biopsy as described in Methods. HFF2 fibroblasts were purchased from ATCC (SCRC1041TM)

Authentication

HFF1 fibroblasts were authenticated by determining fibroblast marker expression. HFF2 were not authenticated.

March 2021

Mycoplasma contamination	Negative for mycoplasma contamination
Commonly misidentified lines (See ICLAC register)	*Name any commonly misidentified cell lines used in the study and provide a rationale for their use.*

Animals and other organisms

Policy information about studies involving animals; ARRIVE guidelines recommended for reporting animal research

Laboratory animals	As described in the methods section, NSG immunocompromised mice were purchased from Jackson Laboratories and maintained at the animal barrier facilities of the University of Barcelona.
Wild animals	No wild animals were used.
Field-collected samples	No field-collected samples were used.
Ethics oversight	All animal procedures were approved by the Animal Ethics/Research Committee of the University of Barcelona.

Note that full information on the approval of the study protocol must also be provided in the manuscript.

Human research participants

Policy information about studies involving human research participants

Population characteristics	Human data is not included in the submitted manuscript. But detailed information can be provided upon request as a supplemental table.
Recruitment	We did not recruit human participants. All human islet samples are isolated from cadaveric donors at islet isolation centers.
Ethics oversight	Experiments involving human islets were performed in agreement with the local ethic committee (CHU, Montpellier) and the institutional ethical committee of the French Agence de la Biomédecine (DC Nos. 2014-2473 and 2016-2716).

Note that full information on the approval of the study protocol must also be provided in the manuscript.
